# *IFNAR2*-dependent gene expression profile induced by IFN-α in *Pteropus alecto* bat cells and impact of *IFNAR2* knockout on virus infection

**DOI:** 10.1371/journal.pone.0182866

**Published:** 2017-08-09

**Authors:** Qian Zhang, Lei-Ping Zeng, Peng Zhou, Aaron T. Irving, Shang Li, Zheng-Li Shi, Lin-Fa Wang

**Affiliations:** 1 Key Laboratory of Special Pathogens, Wuhan Institute of Virology, Chinese Academy of Sciences, Wuhan, China; 2 Programme in Emerging Infectious Diseases, Duke–National University of Singapore Medical School, Singapore, Singapore; 3 Programme in Cancer and Stem Cell Biology, Duke–National University of Singapore Medical School, Singapore, Singapore; Institut Pasteur, FRANCE

## Abstract

Bats are important reservoirs of many viruses, which are capable of infecting the host without inducing obvious clinical diseases. Interferon and the downstream interferon regulated genes (IRGs) are known to act as the first line of defense against viral infections. Little is known about the transcriptional profile of genes being induced by interferon in bats and their role in controlling virus infection. In this study, we constructed *IFNAR2* knockout bat cell lines using CRISPR technology and further characterized gene expression profiles induced by the most abundant IFN-α (IFN-α3). Firstly, we demonstrated that the CRISPR/Cas9 system is applicable for bat cells as this represents the first CRIPSR knockout cell line for bats. Our results showed the pleiotropic effect of IFN-α3 on the bat kidney cell line, PaKiT03. As expected, we confirmed that *IFNAR2* is indispensable for IFN-a signaling pathway and plays an important role in antiviral immunity. Unexpectedly, we also identified novel *IFNAR2*-dependent IRGs which are enriched in pathways related to cancer. To our knowledge, this seems to be bat-specific as no such observation has been reported for other mammalian species. This study expands our knowledge about bat immunology and the cell line established can provide a powerful tool for future study into virus-bat interaction and cancer biology.

## Introduction

Most emerging and re-emerging infectious diseases are zoonotic. Bats are generally recognized as one of the most important reservoirs of zoonotic agents and they can carry many viruses without showing obvious clinical symptoms [1–3]. The mechanisms of how bats successfully co-exist with various viruses are intriguing and largely remain unknown. In recent years, some interesting findings seem to point to some bat-specific evolutionary events which resulted in several bat-specific functional characteristics not shared by other mammals. Comparative genomic analysis of *Pteropus alecto* and *Myotis davidii* shows an unexpected amount of positively selected genes involved in the DNA damage checkpoint and NF-kB pathways [4]. Ahn et al. found the functions of PYHIN gene family in bats seemed to be uniquely lost by genomic analysis of ten bat species, which may limit excessive inflammatory activation from DNA damage [5]. Although most interferon related genes are highly conserved between bats and other mammalian species, bats exhibit some qualitative and quantitative differences in the innate immune system [6, 7]. *P*.*alecto* only have three functional IFN-α genes, contrary to other mammals which have 7–18. Moreover, *P*.*alecto* IFN-α genes seem to be constitutively expressed in unstimulated tissues and cells, possibly acting as a 24/7 front line defense against infection and potentially other diseases[8].

IFN-α initiates signaling through a heterodimeric transmembrane receptor termed the IFN-α receptor (IFNAR), which is composed of IFNAR1 and IFNAR2 subunits. All IFNs can rapidly bind to high-affinity IFNAR2 subunit, and then recruit the low-affinity IFNAR1 chain to form an active ternary complex in human [9]. This process also brings into proximity the intracellular signaling adaptors Tyk2 (with IFNAR1) and JAK1 (with IFNAR2). JAK phosphorylate each other and further activate signal transducer and activator of transcription 1 (STAT1) and STAT2 molecules, leading to their dissociation, dimerization and finally binding of these molecules to IRF9 to form the ISG factor 3 (ISGF3) complex. This complex then translocates to nucleus, binds to IFN-stimulated response elements (ISRE) in interferon regulated gene (IRG) promoters and drives the activation of IRGs transcription [10]. IRGs encode direct antiviral effectors or molecules with the potential to positively and negatively regulate IFN signaling and other host responses.

CRISPR systems are adaptable immune mechanisms used by many bacteria to protect themselves from foreign genetic elements, such as viruses or plasmids. Among them, the CRISPR/CRISPR-associated protein 9 (CRISPR/Cas9) is the most developed and used system for creating double-strand DNA breaks in any genomic location of interest by a customizable short RNA guide [11]. Repair of Cas9 lesions by non-homologous DNA end joining (NHEJ) mechanism can introduce indel mutations within a coding exon, leading to frameshift mutations and premature stop codons, thus inactivate the function of specific genes. The CRISPR/Cas9 system has been successfully applied to different kinds of species, including human, mice, pig, cow, zebrafish, drosophila and *caenorhabditis elegans* [11]. While it is expected to also work in bats, this study represents the first trial in any bat species.

Although our previous studies demonstrated that *P*.*alecto* IFN-α plays an important role in antiviral immunity, no comprehensive gene profile studies have been conducted to examine whether the expression profile of genes induced by IFN-α in this species will be similar to that in other mammals or whether there will be some bat-specific genes or pathways. As a first step towards a genetic and functional profiling of IFN-α induced genes in *P*.*alecto*, we blocked the interferon pathway by knocking out the *IFNAR2* gene in a *P*.*alecto* kidney cell line using CRISPR technology. Genome-wide transcriptomics and viral infection were then conducted for both the wild-type and knockout cell lines to establish genetic and functional characterization of IFN-α induced genes.

## Materials and methods

### Guide RNA (gRNA) design and plasmid construction

Exon sequence close to 5’-end of *IFNAR2* gene was submitted to online software (http://tools.genome-engineering.org) to obtain potential gRNA targets [11]. The top hits were further subjected to blast with *P*.*alecto* genome to exclude unwanted off-target effects. The final two gRNA sequences with both high score and specificity were chosen for plasmid construction. The pSpCas9 (BB) -2A-GFP plasmid was used as a vector for the current study following previously published protocol [11].

### Single cell screening and validation

PaKiT03 cells, which are immortalized kidney cell lines from *P*.*alecto* [12], were seeded onto 6-well plates at 8×10^5^ cells/well and transfected with 1.5 μg plasmid using Lipofectamine 3000 (Life Technologies, Carlsbad, CA, USA) following manufacturer’s recommendation. Two days after transfection, cells were sorted using FACSAria III (BD Biosciences, San Jose, CA). GFP-positive clones were collected and plated onto 96-well plates at a concentration of approximately 2 cells/well. One week later, the single colony cells were selected for further validation using fluorescent capillary gel electrophoresis as follows: genomic DNA of single colony cells were extracted using the QuickExtract solution (Epicentre, Madison, WI, USA). Primers used for fluorescent PCR were designed spanning gRNA sequence with an amplicon size of around 350 bp. The forward primer was covalently linked with the 6-FAM or HEX at the 5′end. The HEX-labelled primer was used to amplify parental wild-type gDNA as control, while 6-FAM-labelled primer was used for targeted clones with potential indel mutations. PCR was conducted with Taq DNA polymerase (QIAGEN, Germany) and PCR products were analyzed using capillary gel electrophoresis as described previously [13].

### RNA extraction and quantitative RT-PCR

RNA was extracted using EZNA total RNA kit (Omega Bio-tek, Norcross, GA, USA). Extracted RNA (500 ng) was subsequently used for cDNA synthesized using QuantiTect^®^ Reverse Transcription Kit (QIAGEN, Germany). Reactions of qPCR were setup using the SensiFAST^™^ SYBR No-ROX Kit (Bioline, London, UK) and analyzed on the CFX96 Touch^™^ Real-Time PCR Detection System (Bio-Rad, CA, USA) under the following cycling condition: 95°C for 5 min, followed by 40 cycles of 95°C for 5 s and 57°C for 30 s, and ending with a melt profile analysis. The fold change in mRNA expression was determined using the 2^-ΔΔCt^ method relatively to the values in mock samples, after normalization to housekeeping gene SNRDP3 [14].

### Western blot analysis

Cells were resuspended in lysis buffer containing protease inhibitors and phosphatase inhibitors. Proteins were separated in 10% SDS-PAGE gels, followed by transfer onto PVDF membranes (Millipore, Billerica, MA, USA). The membranes were incubated with primary antibody overnight at 4°C, followed by incubation with secondary antibody for 1 h at room temperature. Antibodies/dilution used in this study were as follows: anti-Mx1 (ab95926, Abcam)/1:1000, anti-STAT1 (9172S, Cell Signaling Technology)/1:500, anti-pSTAT1 (7649S, Cell Signaling Technology)/1:500 and anti-Actin (A2228, Sigma)/1:5000.

### RNAseq analysis

Total RNA was checked using the RNA 6000 LabChip Kit on the Agilent Bioanalyzer (Agilent Technologies, Palo Alto, CA). RNAseq libraries were prepared using Illumina Tru-Seq Stranded Total RNA with Ribo-Zero Gold kit following the manufacturer’s instructions (Illumina, San Diego, California, USA). Libraries were validated with an Agilent Bioanalyzer (Agilent Technologies, Palo Alto, CA), diluted and applied to an Illumina flow cell using the Illumina cBOT system. Sequencing was performed on an Illumina HiSeq 3000 sequencer at the Duke-NUS Genome Biology Facility with the paired-end 150-bp read option.

After trimming and cleaning for quality assurance, all reads were mapped to the *P*.*alecto* reference genome (NCBI genome database: ASM32557v1) with Bowtie and performed RSEM abundance estimation [15]. EBseq and edgeR were used to detect genes that differentially expressed after IFN-α3 treatment [16, 17]. The cut-off for differentially expressed genes (DEGs) was set at >2-fold change and p-value ≤ 0.05. These DEGs were compared with known interferon regulated genes (IRGs) in human and mouse which are accessible from an online open access database INTERFEROME (http://www.interferome.org/interferome/home.jspx) [18]. DEGs lists were further processed with Ingenuity Pathway Analysis (IPA) software (Ingenuity Systems Inc., Redwood City, CA) for enrichment and pathway analysis.

### IFN-α3 treatment

IFN-α3 was expressed in HEK293T cells transiently transfected with the pCAGGS/FLAG–IFN-α3 plasmid and purified as previously described [8]. The concentration of protein was measured by Nanodrop. Both wild-type PaKiT03 and *IFNAR2* knockout (KO) cell lines were treated with 100 ng/ml IFN-α3 for 6h. Cells were then collected into TRK lysis buffer (Omega Bio-tek, Norcross, GA, USA) for RNA extraction.

### Virus infection

Wild-type or *IFNAR2* KO cells were seeded onto 6-well plates at 1×10^6^ cells/well. After 8 h, cells were completely attached to the plate and infected with H1N1 influenza A virus (A/NWS/33, ATCC) at MOI of 0.1, followed by a further 2-h incubation. Virus-containing supernatants were then replaced with fresh DMEM medium and incubated for 48 h. Culture supernatants were harvested for virus titration by plaque assay in BHK cells. For IFN-α3 treatment, cells were incubated for 2 h with IFN-α3 (100 ng/ml) before virus infection.

## Results

### Functional knockout of IFNAR2 using CRISPR

**Fig 1:**
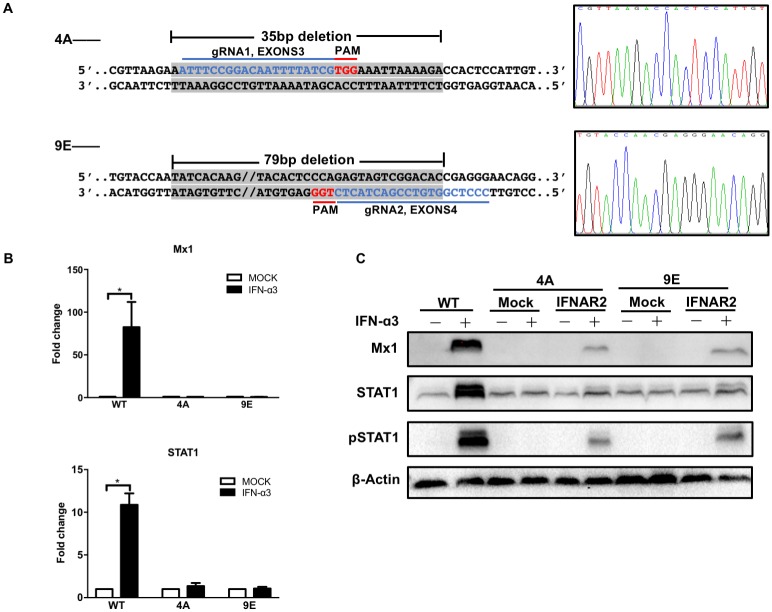
Verification of *IFNAR2* KO in two cell lines obtained from two independent gRNAs. (A) Sanger sequencing was performed to validate the location and nature of the deletion events. Left: The location of gRNA and PAM motif are given in blue and red, respectively. The deletion regions are highlighted in gray. Right: Chromatogram of DNA sequence spanning the deletion region. Quantitative RT-PCR (B) and western blot (C) analyses were performed to confirm the functional phenotype of the clones.

Two different gRNA sequences located in exons3 (gRNA1) and exons4 (gRNA2), respectively, of the *IFNAR2* gene were used for CRISPR knockout to reduce the off-target probability (Fig 1A). BLAST search against the *P*.*alecto* genome using the 12-bp gRNA seed sequences revealed 5 potential off-targets sites for each of the two gRNAs, but none was followed by the protospacer-adjacent motif (PAM) sequence-NGG.

Fluorescent PCR coupled with capillary gel electrophoresis was employed to detect insertion/deletion (indel) mutations in targeted cells following published method [13]. This technique can accurately and efficiently predict the number of nucleotide(s) inserted or deleted in a high throughput manner, and more importantly it can distinguish heterozygous mutants from homozygous mutants. Initial analyses indicated that 12 out of 48 clones (25.0%) showed a shift in fragment size with gRNA1 and 9 out of 32 (28.1%) with gRNA2. Representative clones were then verified using Sanger sequencing, all confirming findings from fluorescent capillary electrophoresis (S1 Table). Finally, we selected clones 4A (gRNA1) and 9E (gRNA2) with 35-bp and 79-bp deletion in the exon region, respectively, for further study (Fig 1A). Both 4A and 9E introduced homozygous changes which cause frameshifting (Fig 1A).

To exclude potential off-target effect in these two clones, we conducted PCR to amplify and sequence the genome regions with predicted potential off-targets. No indel mutations were detected in any of the sites for either clone. We are therefore confident that chance the off-target effect is slim although we can never rule out other potential off-target sites which were not predicted as the assembly of the genome is not perfect.

Due to the lack of suitable antibody for *P*.*alecto IFNAR2*, it was not possible to validate the KO by western blot. We therefore examined downstream gene expression upon treatment by qPCR and western blot to functionally confirm *IFNAR2* KO (Fig 1B and 1C). The choice of IFN-α3 as the subtype for this study was based on previous observations that IFN-α3 is the most abundant subtype in various bat tissues [8]. As shown, the expression of Mx1 and STAT1 were completely abolished in 4A and 9E, at both the mRNA level (6h) and the protein level (24h). Phosphorylated STAT1 (1h) could only be detected in wild-type PaKiT03 cells and not in 4A or 9E cells. Moreover, the phenotype could revert upon IFN-α3 treatment in 4A and 9E cells transfected with *IFNAR2*, which could further exclude off-target effects of CRISPR (Fig 1C).

### Differentially expressed genes induced by IFN-α3 in wild-type cells and complete abrogation of IFN signaling in IFNAR2 KO cells

For the wild-type PaKiT03 cells, a total of 578 genes were up-regulated and 105 down-regulated after IFN-α3 treatment based on significant difference criterion with Log2FC >1 and p-value ≤ 0.05 (Table 1 and S2 Table). Of these 578 genes, 418 are known IRGs, and the remaining 160 seemed to be *P*.*alecto* specific IRGs, which were tentatively defined as unknown IRGs in this study. Likewise, among the 105 down-regulated genes, 54 were known IRGs and the other 51 were unknown IRGs (S3 Table). It was noted that fold changes of down-regulated genes were quite small, the top 12 genes were decreased around 4.0–12.3-fold, and 6 of them were unknown IRGs.

**Table 1:** Number of DEGs induced by IFN-α3 in wild-type PaKiT03 and *IFNAR2* knockout cell lines.

Cells	Up-regulated			Down-regulated		
	All	Known IRG*	Unknown IRG	All	Known IRG	Unknown IRG
WT	578	418	160	105	54	51
4A	0	0	0	0	0	0
9E	0	0	0	0	0	0

*IRG: Interferon regulated genes. Genes that recorded in INTERFEROME database were recognized as known IRG.

When the same analysis using EBseq software was conducted on the KO cells based on the same significant difference criterion, no genes were up- or down-regulated by IFN-α3. To further validate this finding, we used a different software, edgeR, to re-analyze the data using the same criterion. Surprisingly, the findings were almost identical to the EBseq data with the exception of only1-2 genes found to be up- or down-regulated with 2-fold change. Moreover, no common DEGs were observed between the two KO cell lines (S4 Table). This result indicated the *IFNAR2* KO has completely abrogated IFN-α3 signaling in PaKiT03 cells.

### Functional enrichment analysis

**Fig 2:**
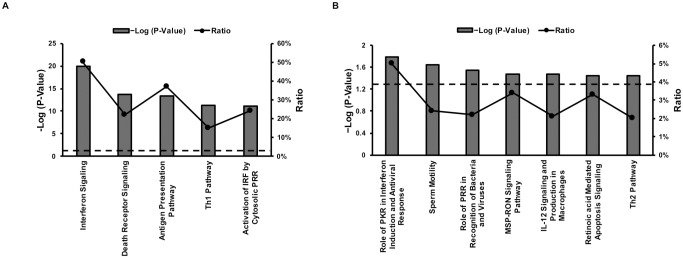
Canonical pathways analysis for the up-regulated total IRGs (A) and the up-regulated unknown IRGs (B) using IPA software. Statistical significance is represented by -log (P-Value), and values exceeding 1.30 (indicated by the dotted lines) are considered significant (P<0.05). The ratio represents the percentages of genes enriched to the total number of genes in each category.

To explore whether the IRGs identified from the above study share specific functional features, we examined canonical pathway analysis by performing Fisher’s exact test in the IPA system (S5 Table). The 5 most significantly enriched pathways are shown in Fig 2A. As expected, the terms representing interferon signaling were most significant (p<0.001). The second pathway was associated with death receptor signaling, including genes involved in regulation of I-κB kinase/NF-κB signaling (IKBKE, RIPK1) and apoptotic signaling (TNFSF10, FAS) [19, 20]). Notably, among this category, a large number of genes belong to the PARP family. These genes can modify various nuclear proteins by poly-ADP-ribosylation, which is required for regulation of cell differentiation and proliferation, gene transcription, and tumor transformation [21]. In addition, genes involved in the Th1 pathway were highly enriched as well, such as CD274, CD40, LGALS9 [22, 23, 24].

Next, the up-regulated unknown IRGs were analyzed using the IPA software (S5 Table). Surprisingly, it contained genes (such as RNASEL and APAF1) that are involved in interferon induction and antiviral response by interaction with protein kinase R (PKR). RNASEL, together with PRKCD and IL12A could also have an effect on the role of pattern-recognition receptors (PRR) in the recognition of bacteria and viruses. In addition, these unknown IRGs are implicated in apoptosis (APAF1, PARP16) and Th2 signaling (BHLHE41, HAVCR1, IL12A) (Fig 2B) [25, 26, 27]. Intriguingly, when IPA analysis was restricted to *P*.*alecto* specific IRG genes for particular biological and disease processes, most of them were enriched in cancer and organismal injury/abnormalities categories (Table 2 and S6 Table).

**Table 2:** The top-ranked diseases enriched by IPA analysis for the unknown IRGs.

Diseases and Disorders	P-value* range	No. (%) size (bp)
Cancer, Organismal Injury and Abnormalities;	4.31E-06	99 (61.9)
Cell Morphology, Cell-To-Cell Signaling and Interaction, Cellular Assembly and Organization;	2.33E-05	2 (1.3)
Auditory and Vestibular System Development and Function, Organ Morphology, Organismal Development;	6.96E-05	2 (1.3)
Reproductive System Disease.	1.39E-04	2 (1.3)

*P-value indicated the probability of the association between the genes in the dataset and disease terms.

The IFN-stimulated response element (ISRE) located in the promoter region of IRGs is required for IFN-inducible transcription. To explore whether these unknown IRGs were regulated by IFNs directly, 32 unknown IRGs were selected for promoter analysis using TRANSFAC database by focusing on the 1,000-bp sequence region upstream of the ATG start codon of a given IRG ORF. We also analyzed the corresponding region of human genes for comparison and found 14 versus 4 potential ISREs in bat and human genes, respectively (S7 Table). This result suggested that these unknown IRGs in bats could be regulated by interferon in both direct and indirect manners.

### IFN-α-mediated antiviral effect is IFNAR2-dependent

**Fig 3:**
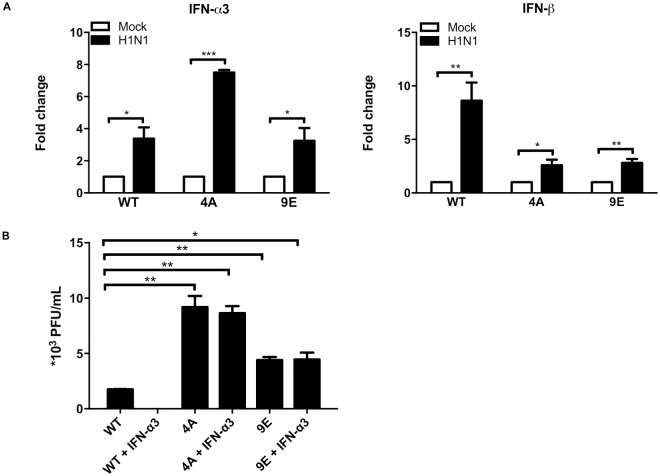
Effect of *IFNAR2* KO on H1N1 infection in bat cells. Cells were treated with IFN-α3 for 2 hrs before infected with H1N1 at MOI of 0.1. Cells and Culture supernatants were harvested at 48 hrs post infection. Gene expression was determined by measuring mRNA level using qPCR (A) and the data were normalized against the expression level of the housekeeping gene SNRPD3. Virus titers were determined by plaque assay in BHK cells (B). Error bars indicate standard deviations from three independent experiments.

From the result above, we know that *IFNAR2* is indispensable for activation of downstream anti-viral genes in response to IFN-α3. We also know from our previous work that IFN-α3 has antiviral activity[8]. To further confirm the role of *IFNAR2* signaling in IFN-α3-mediated anti-viral response, we compared influenza virus H1N1 replication in wild-type and *IFNAR2* KO cell lines in the absence and presence of exogenous IFN-α3. As shown in Fig 3, influenza virus H1N1 was able to induce both IFN-α and IFN-β in wild-type PaKiT03 (Fig 3A), which is in accordance with studies of H1N1 infection in cell lines of other mammalian species [28, 29]. We also found that H1N1 replicated more efficiently in *IFNAR2* KO cell lines, indicating that endogenous interferons have some protective effect from H1N1 infection through an *IFNAR2*-dependent pathway (Fig 3B). This was further confirmed by adding exogenous IFN-α3 before virus infection, demonstrating that H1N1 infection was completely abrogated in wild-type cell lines while there was no change in H1N1 infected *IFNAR2* KO cell lines (Fig 3B).

## Discussion

In this study, we successfully applied CRISPR technology in making *IFNAR2* KO bat cell line and further demonstrated the functional loss of *IFNAR2* in IFN-α3-mediated antiviral immunity. To our knowledge, this is the first successful application of CRISPR to any bat species. While this may be considered trivial as the technology has been applied to many different mammalian species [30, 31], it is nevertheless an important observation in the context of the potential uniqueness of bat DNA damage repair system [4].

In CRISPR technology, non-homologous end joining (NHEJ)-mediated repair of Cas9-generated double-stranded breaks (DSBs) is the key to make a null allele in any genes of interest. While the essential genes involved in NHEJ pathway, such as DNA-dependent protein kinase (DNA-PKc), Ku80 and Ligase 4 (LIG4), were found to be under positive selection in bats [4]. Therefore, it is hypothesized that a more efficient DNA damage repair system is evolved to better cope with the negative effects of reactive oxidative species (ROS), one of the major causes of DSBs, which are produced in high amount during much higher metabolism during flight [4]. In this context, it has been questioned whether CRISPR technique can work equally well in bats as in other mammals.

Although this was only one example, we have demonstrated the CRISPR can be used with bat cells. Since then, we have made several other knockout bat cell lines (to be published elsewhere). Strikingly, in our study, the deletion in both bat cell lines seems to be larger (15-131bp) than what has been reported previously in other species with indel of 1–14 bps [32]. It remains to be seen whether the larger than usual indels is a general trend for bats of different species, and if so, whether this is related to the supposed unique DNA damage repair system in bats [4].

In our study, up-regulated genes were induced at much higher levels compared with down-regulated genes after IFN-α treatment. This may be due to the timing of sampling, as the half-life of down-regulated genes is generally shorter than those of up-regulated genes [33]. This was also the case in another study which examined the gene expression profile in endothelial cells after 5 h of activation by IFN-α [34]. Further studies are needed to get more precise data about down-regulated genes.

IFN activities can be divided into robust and tunable, based on their timing of induction and their sensitivity to the receptor and IFNs [10]. IFN-α3, the most abundant IFN-α in *P*. *alecto* tissues, seemed to have pleiotropic effect on PaKiT03 cells. It could induce not only robust response of genes associated with anti-virus and antigen presentation processes (such as Mx1/2, OAS1-3, HLA-DRB1, CIITA), but also tunable responses which strongly depend on the cellular context. Tunable genes normally are related to cytokine and chemokine activities, inflammatory and anti-proliferative activities [35], such as CXCL11, IDO1, TNFSF13B which were identified in this current study. Such diversity is believed to be important for efficient crosstalk between innate immunity and other signaling pathways. For examples, a number of genes induced by IFN are involved in Th1 activation pathway, thus bridging innate and adaptive immunity. This is not found in other non-immune cells [33, 36]. Genes related to DNA damage regulation were also induced, including CDKN1A, SP100, PML and SIRT1 [37, 38, 39].

The crosstalk between these pathways may play a more important role in bats than in other terrestrial mammals. As flying mammals, bats are exposed to high metabolic “attack” during daily flights and are likely to be exposed to greater variety of pathogens as a result of their long-distance travel ability. In this context, it is interesting to note from this preliminary study that bats seem to generate a broader spectrum of IFN inducible genes. On the other hand, *P*. *alecto* share identical negative regulatory mechanism as other mammals, with SOCS1 and USP18 highly induced to avoid toxic consequences of excessive signaling [40, 41].

One of the important discoveries made in this preliminary study is the large number of the so-called “unknown” IRGs (160 of 578 or 27.7%) in *P*. *alecto*, which have not been previously identified in other species. RNASEL, a component of the well-known interferon-regulated 2-5A system, was incorporated in this group [42]. This finding suggest that virus sensing could be amplified in bats compared with other species. Significantly, IPA analysis indicated that 61.9% of them are implicated in cancer pathways. IFNs are known for their antitumor effects, as IRGs are broadly expressed across various tumor types and contribute to chemotherapy and radiation therapy [43]. As bats are believed to have lower rates of tumorigenesis [3, 44], our current findings are likely to shed new light on the role of interferon in cancer immunity.

Despite the fact that IFN-β can selectively activate a group of non-STAT regulated genes in the absence of *IFNAR2*, whether IFN-α can also be functional in cells lacking *IFNAR2* remains unknown [45]. In our study, we unequivocally demonstrated that, at least for *P*. *alecto* IFN-α3, IFN-α pathway is fully dependent on *IFNAR2* by transcriptomics-based analysis, suggesting that *IFNAR2* is indispensable for defending against virus infection in bats. On the other hand, our result implied that the binding affinities of bat IFN-α toward *IFNAR1* and *IFNAR2* are quite different [46]. Further studies are needed to validate this.

## Supporting information

S1 Table Results of fluorescent PCR-capillary gel electrophoresis in wild-type PaKiT03 and *IFNAR2* KO cells. Green peaks indicate fragments obtained from wild-type PaKiT03 cells using HEX-labeled primers, and act as an internal size control. Blue peaks indicate fragments obtained from knockout cells using 6-FAM-labeled primers. The numbers given in each plot represent the sizes of each fragment and those in parentheses are the calculated difference in size (in base pairs) with respect to individual wildtype fragments.
(PDF)

S2 Table List of differentially expressed genes induced by IFN-α3 in wild-type PaKiT03 cells. (XLSX)

S3 Table List of unknown IRGs induced by IFN-α3 in wild-type PaKiT03 cells. (XLSX)

S4 Table Differentially expressed genes detected in *IFNAR2* KO cell lines using edgeR software. (PDF)

S5 Table List of canonical pathways analysis for the up-regulated IRGs using IPA software. (XLSX)

S6 Table List of diseases and biological functions analysis for the up-regulated unknown IRGs using IPA software. (XLSX)

S7 Table Analysis of ISRE within the promoter region of unknown IRGs by TRANSFAC database. (PDF)
