# Correction: *IFNAR2*-dependent gene expression profile induced by IFN-α in *Pteropus alecto* bat cells and impact of *IFNAR2* knockout on virus infection

**DOI:** 10.1371/journal.pone.0191578

**Published:** 2018-01-17

**Authors:** Qian Zhang, Lei-Ping Zeng, Peng Zhou, Aaron T. Irving, Shang Li, Zheng-Li Shi, Lin-Fa Wang

There is an affiliation missing for the first author. Qian Zhang should also be affiliated with: University of Chinese Academy of Sciences, Beijing, China.
