# Supplementary figures and images for: IFNAR2-dependent gene expression profile induced by IFN-α in Pteropus alecto bat cells and impact of IFNAR2 knockout on virus infection
Source: PLoS One. 2017 Aug 9;12(8):e0182866. doi: 10.1371/journal.pone.0182866 (PMC5549907; doi:10.1371/journal.pone.0182866)

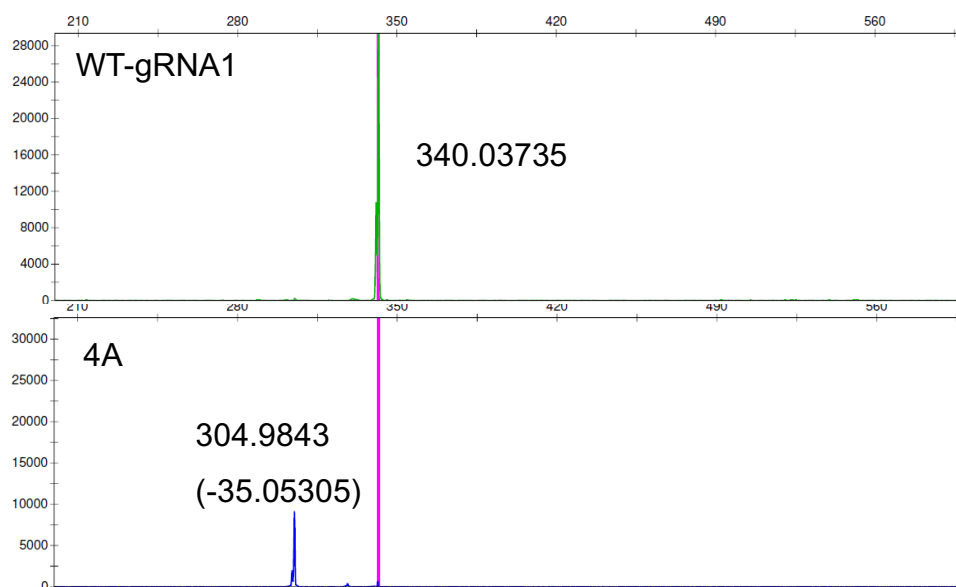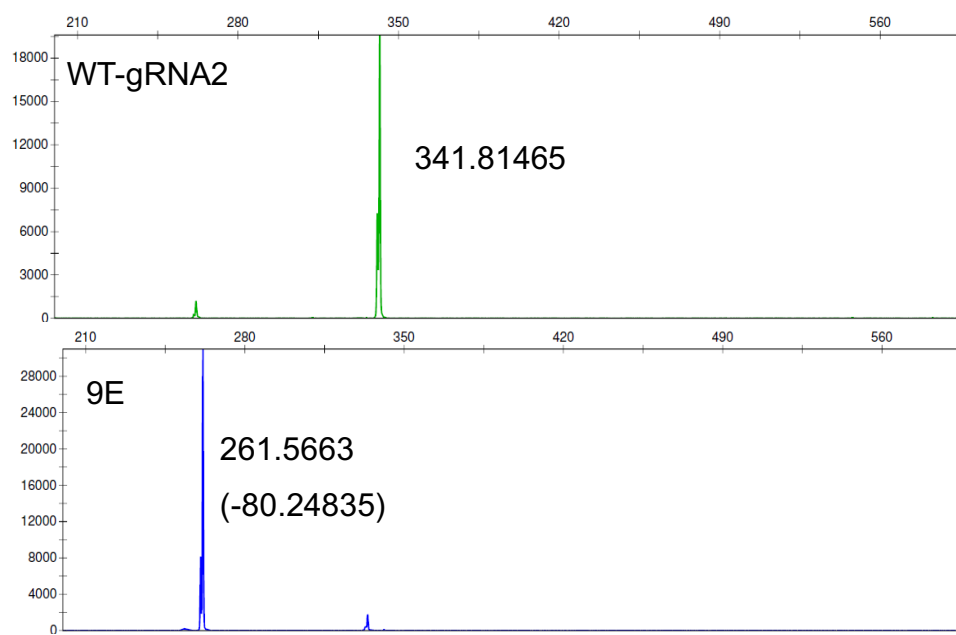

Supplement: S1 Table — Green peaks indicate fragments obtained from wild-type PaKiT03 cells using HEX-labeled primers, and act as an internal size control. Blue peaks indicate fragments obtained from knockout cells using 6-FAM-labeled primers. The numbers given in each plot represent the sizes of each fragment and those in parentheses are the calculated difference in size (in base pairs) with respect to individual wildtype fragments. (PDF) [file pone.0182866.s001.pdf]
