# Supplementary material for: IFNAR2-dependent gene expression profile induced by IFN-α in Pteropus alecto bat cells and impact of IFNAR2 knockout on virus infection
Source: PLoS One. 2017 Aug 9;12(8):e0182866. doi: 10.1371/journal.pone.0182866 (PMC5549907; doi:10.1371/journal.pone.0182866)
Supplement: S4 Table — (PDF) [file pone.0182866.s004.pdf]

Cells	Genes	Log2FC	P-value
4A	PLPPR4	1.010	0.0056
	LOC102880632	-1.041	0.0033
	LOC102893089	-1.027	0.0003
9E	RGMA	1.006	0.0018
	CCR10	-1.149	0.0005
