# Supplementary material for: IFNAR2-dependent gene expression profile induced by IFN-α in Pteropus alecto bat cells and impact of IFNAR2 knockout on virus infection
Source: PLoS One. 2017 Aug 9;12(8):e0182866. doi: 10.1371/journal.pone.0182866 (PMC5549907; doi:10.1371/journal.pone.0182866)
Supplement: S7 Table — (PDF) [file pone.0182866.s007.pdf]

Species	Genes	Predicted ISRE
P.alecto	RNASEL	GAAACGAAA
	TTC31	GAAAGTGAAA
	SIRT1	GAAACTGAAA
	CBLN3	GAAACTGAAA
	SMAGP	GAACTGAAA
	IL12A	GAAAGCGAAA
	TCEANC2	GAACTGAAA
	FAM115C	GAAACGAAA
	IL17RC	GAAACGAAAG
	PITX2	GAAATGAAA
	FOXS1	GAAAGAGAAA
	PRRG2	GAAATCGAAA
	AFAP1L2	GAAATGAAA
	SLC24A1	GAAAGAGAA
Human	SMAGP	GAACCGAAA
	IL12A	GAAAGCGAAA
	TCEANC2	GAATTGAAA
	SLC24A1	GAAGGGAAA
